# The Reality of a Head-Mounted Display (HMD) Environment Tested via Lightness Perception

**DOI:** 10.3390/jimaging10020036

**Published:** 2024-01-29

**Authors:** Ichiro Kuriki, Kazuki Sato, Satoshi Shioiri

**Affiliations:** 1 Graduate School of Science and Engineering, Saitama University, Saitama 338-8570, Japan; 2 Graduate School of Information Sciences, Tohoku University, Sendai 980-8579, Japan; 3 Research Institute of Electrical Communication, Tohoku University, Sendai 980-8570, Japan

**Keywords:** lightness perception, lightness constancy, virtual reality, head-mounted display

## Abstract

Head-mounted displays (HMDs) are becoming more and more popular as a device for displaying a virtual reality space, but how real are they? The present study attempted to quantitatively evaluate the degree of reality achieved with HMDs by using a perceptual phenomenon as a measure. Lightness constancy is an ability that is present in human visual perception, in which the perceived reflectance (i.e., the lightness) of objects appears to stay constant across illuminant changes. Studies on color/lightness constancy in humans have shown that the degree of constancy is high, in general, when real objects are used as stimuli. We asked participants to make lightness matches between two virtual environments with different illuminant intensities, as presented in an HMD. The participants’ matches showed a high degree of lightness constancy in the HMD; our results marked no less than 74.2% (84.8% at the maximum) in terms of the constancy index, whereas the average score on the computer screen was around 65%. The effect of head-tracking ability was confirmed by disabling that function, and the result showed a significant drop in the constancy index but that it was equally effective when the virtual environment was generated by replay motions. HMDs yield a realistic environment, with the extension of the visual scene being accompanied by head motions.

## 1. Introduction

Head-mounted displays (HMDs) are becoming more popular as a tool with which to present a virtual visual environment. They have been used in various scenarios, including entertainment, engineering, and for scientific research purposes [1,2,3]. In general, real objects yield high precision in the perception of an object’s surface properties, such as color and lightness constancy [3,4,5,6,7,8,9,10,11,12,13,14,15,16,17,18,19,20,21,22,23]. Lightness constancy is a perceptual phenomenon, in which the perception of object reflectance is maintained constantly under changes in illuminant strength. When a white object is placed under bright illumination or dim illumination, where the illuminant strength changes from 10 to 1, the white object reflects 10 times less light under dim illumination than under bright illumination. If the light intensity is directly related to the perception of object surface property, the white object under the dimmer illuminant should be perceived as “gray”, with 1/10 reflectance. However, we can perceive the reflectance (i.e., the lightness) of an object as relatively stable for real objects in a real environment. Thus, we can assume that the estimation of light intensity is relatively stable in a real environment, enabling us to discount changes in illuminant intensity when perceiving an object’s surface properties. Color constancy is known to be an analogous phenomenon that occurs with color [4,5,6,7,8,9,10,11,12,13,14,15,16,17,18,19,20,21,22,23] and it has been intensively studied for implementation as an image processing algorithm [5]. The lightness constancy is one aspect of color constancy in the lightness scale. This lightness is one of the most basic perceptions regarding the surface property of materials [4]. The perception of color/lightness constancy is known to emerge in infants of less than a year old [23], which implies that it is one of the most fundamental properties of the human visual system [24,25,26]. It also affects the appearance of an object’s color and material properties on computer screens [12,27].

Color/lightness constancy has been studied in many previous studies under various apparatus and evaluation conditions [4]. The degree of constancy has been evaluated by a quantitative measure, which is defined, traditionally, as a colorimetric shift in color/luminance that yields the same color/lightness perception, normalized to the colorimetric shift in color/luminance of a target across illuminant changes [4]. If the compensation of the human visual system is 100 percent, the degree of shift in color/luminance of a light that yields the same perception would be identical to the colorimetric shift across the illuminant changes. This ratio is called the constancy index or “Brunswik” ratio (in short, “BR”; see also Equation (1) in Section 2, *Materials and Methods*).

It is considered, in general, that the degree of color/lightness constancy is higher in realistic viewing conditions. The constancy indices/BRs ranged from about 20% to over 90% [6,7,8,9,10,11,12,13,14,15,16,17,18,19,20,21,22] in past studies on color/lightness constancy, depending on the experimental conditions. In general, the degree of color/lightness constancy is higher in experiments with real objects [9,10,11,12,13,14,15,16] than that conducted on a computer screen with a two-dimensional array of colors [6,7]. A review article by Foster (2011) [4] on color constancy has precisely re-evaluated the constancy index and BR for representative studies on color constancy, summarizing them with the experimental conditions in a table (Table 1 in Foster, 2011 [4]). The average BRs for two-dimensional displays were 65% and those for the real objects were 80%. However, some studies conducted on computer screens also yielded a higher degree of color constancy [17,18,19,20,21,22]. These studies used a three-dimensional cue [17,18] or a larger size of adapting field, presented as real objects [19,20], or used a color-naming method [22]. The common factor in those experimental conditions that yielded a high degree of constancy index was the richness of the clue used to recognize the illuminant conditions [17,18,19,20,21,22], regardless of whether the stimuli were real objects or simulated surfaces on computer screens. In fact, a larger field size surrounding the test stimulus (120 deg) yielded a much higher degree of constancy than a smaller field size (20 deg), even among those studies using a real object as a stimulus [14]. 

However, the use of actual objects limits the flexibility and precision of visual stimulus control in scientific studies on visual perception. Computer monitors also have their drawbacks, e.g., in the field of view (FoV) of a flat screen, which can only display around 20–30 deg in terms of visual angle, resulting in the lack of an immersive feeling in the “environments” presented on the screen, and this could have caused a lower degree of color/lightness constancy. The use of HMDs can overcome this FoV problem, because the FoV is much wider (around 85–90 deg) than normal computer monitors, and they can virtually extend the field of observation by allowing the screen images to track the observer’s head direction.

But how far can it provide an appropriate perception of visual scenes? If the display had a fatal limitation or lack, this would cause flaws when using it in studies. There are some studies that have made attempts to evaluate its performance as a tool to investigate the human visual system [1,2]. To address this issue, we measured lightness perception, which is one of the fundamental properties of object perception in the human visual system. 

Previous studies have reported the effect of the temporal integration of visual information across eye movements on color perception [28,29] and lightness [30] perception, using a flat computer screen. Another study has reported that the chromatic induction effect, caused by the change from a colored surround to an achromatic surface, one of the key factors for color constancy, was affected by eye movements [31]. These studies suggest the importance of temporal integration to an object’s surface property of color/lightness constancy. Considering the head-tracking function of HMDs, this may enable the integration of color/lightness information over time from a wider field of view across the virtual environment, compared with the field of view from eye movements across a fixed computer screen. A recent study reported the degree of color constancy [3] by using an HMD with a score of 80 to 90% in the color constancy index. This high degree of color constancy could have been achieved due to the head-tracking function, which enables viewers to obtain information from a larger field of view through images that change in accordance with head motions. However, to our knowledge, no study has reported the effect of the head-tracking function on color/lightness constancy.

Our reason for investigating lightness perception, not color, was because of the low image quality in the HMD that we used. In general, HMDs use a single lens (made from lightweight and low-cost materials) to enable a wider FoV in a compact package that causes, e.g., transverse chromatic aberration to yield chromatic fringes. The HMD system seems to be designed to compensate for this by modifying the image in each color (R, G, or B) channel, but this occasionally causes unexpected color fringes. At least, for the HMD system we used, the image quality was insufficient for scientific research, which demands strict color calibrations. One of the recent studies published by other groups has elaborated on this to evaluate the quality of chromatic calibration with various measurements [3]. Therefore, we avoided the risk of using color as a measure and focused on the achromatic surface property (lightness) perception in the present study. The HMD system in our study used an OLED (organic electro-luminescent display) panel that yields a higher dynamic range of luminance (more than 1:100,000) than the usual liquid crystal display panels (about 1:1000). The use of an OLED screen was crucial for our study of lightness constancy since this wider dynamic range is known to yield better lightness perception than the standard dynamic-range screen [10,11,27,32,33].

In our study, the quality of lightness perception has been measured in a performance to perceive the correct lightness across different lighting environments: that is, lightness constancy. All the objects in the virtual environment of our study share the same illuminant; therefore, the intensity of the reflected light in the environment will change subsequently in the same proportion. The participants’ tasks in the psychophysical experiments of lightness constancy were to report the lightness of a reflecting surface in an unknown (test) environment, by which the degree of lightness constancy was evaluated. In general, the degree of lightness constancy is high in the case of experimental set-ups that use real objects as stimuli, in comparison to the results obtained using computer monitors [4,5,6,7,8,9,10,11,12,13,14,15,16,17,18,19,20,21,22]. It is considered that real objects hold more clues for the viewer to infer the intensity (or color) of the illuminant in each environment for better-quality compensation by human constancy mechanisms. 

As mentioned previously, the size of the FoV could be a key factor to yield better lightness perception [14]. We also measured the effect of head-tracking by the HMD, which enables viewers to obtain information from a larger field of view through images that change in accordance with their head motions.

## 2. Materials and Methods

The participants were volunteers and comprised eleven healthy graduate/undergraduate students (M: 10, F: 1) with normal or corrected-to-normal visual acuity. All experiments were conducted in a dark room. The study was conducted according to the guidelines of the Declaration of Helsinki, and the experimental procedure was approved by the Ethics Committee of the Research Institute of Electrical Communications, Tohoku University (approval code: RIEC-R1-004). Informed consent was obtained from all the participants involved in the study.

We used an HMD Oculus Rift DK2 (Oculus, Irvine, CA, USA) in the experiment, which has a 93-degree FoV in a horizontal direction and a resolution of 980 × 1080 pixels per eye. It uses an OLED (organic electro-luminescent display) panel that generally yields a wide-range luminance contrast of 1:100,000 or more. We calibrated the luminance of the screen with a spectroradiometer, SR-UL1R (Topcon, Tokyo, Japan), by directly measuring the panel surface after removing the eyepiece lens. The Oculus Rift DK2 has interchangeable lenses for different refractive-power individuals. This was another reason for using this particular HMD in this study, to achieve as precise a screen luminance profile as possible.

**Figure 1:**
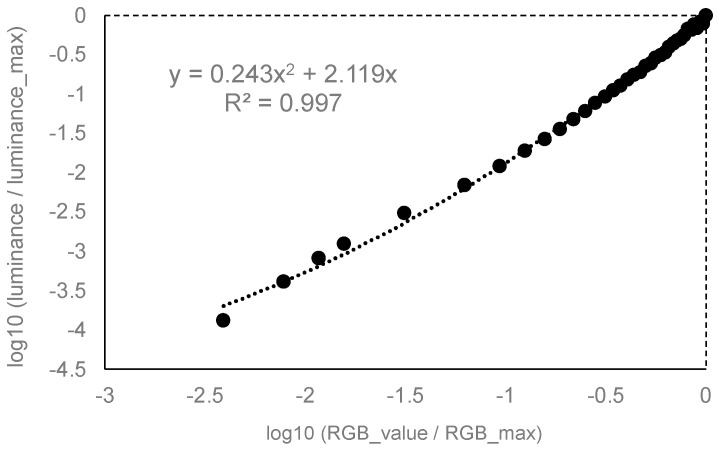
HMD luminance calibration results. The horizontal and vertical axes represent the logarithm of RGB values and the corresponding luminance, both normalized to the maximum. The fitted curve was a second-order polynomial with an intercept at zero. The curve parameters are as shown in the figure. The actual data are in the Supplementary Materials.

The calibration for grayscale (monochromatic) samples on the OLED panel was obtained as follows. The actual values are also shown in the Supplementary Material. The luminance profile with respect to the RGB values was in 8-bit (i.e., 256 steps) resolution. By taking a step of 8 RGB values as a unit, 32 sample points were measured. The luminance for RGB values 1, 2, 3, and 4 were additionally measured to ensure the profile at the lowest luminance range. A program written with the Unity game engine (Unity Technologies, San Francisco, CA, USA) was used to present a rectangular tile covering the entire OLED screen for luminance calibration. The spectroradiometer, SR-UL1R, allowed us to measure lower luminance levels (as low as 0.001 cd/m^2^) [34], and we used luminance values by taking the average of five repeated measurements for each RGB value. To characterize the RGB-luminance profile, we used a previously established method of fitting with a second-order polynomial function to the logarithm of the RGB and luminance values, after normalizing each value to the maximum [35]. As shown in Figure 1, the R^2^ value for the fitting was 0.997. With this method, the luminance for an arbitrary RGB value can be estimated with high precision, without measuring the luminance for all RGB values. The highest luminance at (R, G, B) = (255, 255, 255) was 90.4 cd/m^2^, and the lowest luminance at (R, G, B) = (0, 0, 0) was 1.46 × 10^−5^ cd/m^2^, yielding a luminance dynamic range of 1: 6.20 × 10^6^. The viewing lens was applied during the experiment and the luminous transmittance of the lens was 89.7%. All luminance shown in the following is described for those readings taken at the OLED panel surface (before the viewing lens). 

**Figure 2:**
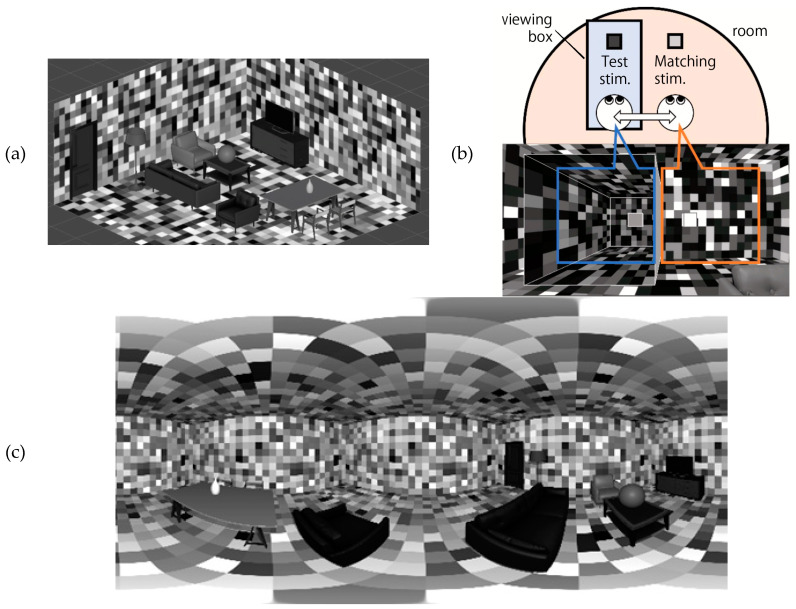
(**a**) The room developed in the study, which was set up in a 3D CG space. The walls are articulated with various lightness patches, arranged in a random order. (**b**) Participants switched their views between the room (right) and viewing box (left) to see the test and matching stimuli in alternation. (**c**) 360-degree image (light field) of the example room image. This conversion was conducted with the Unity script: Camera.RenderToCubemap.

The visual stimulus was an image that simulated a “room,” with walls that were tiled with gray-scale patches (Figure 2a), as rendered by the Unity game engine. The gray tiles were used to control the luminance distribution of the scene and keep it equally uniform in population across the scene. To give ideas about the size of the room to the viewer, model furniture (sofas, cabinets, tables, bottles, etc.) of actual life size was placed in the simulated 3D space (Figure 2a). The room was lit by uniform ambient illumination, wherein the light source was not visible to the participant. The intensity of the illuminance was controlled by scaling the luminance of the room image. All the room images were similar, but the arrangement of the tiles was randomly chosen among 10 variations, and the illuminant intensity was changed simply by scaling the luminance of the room image. This room image was once converted to a 360-degree panoramic image and was then mapped onto a sphere with a radius of 3.0 m in the virtual space around the participant’s head (Figure 2b,c). This conversion was introduced to achieve the precise control of the luminance profile of the scene because shading or mutual reflections between object surfaces are not directly controllable in real-time rendering by Unity. In addition, participants in this study did not walk around; they were sitting on a chair and only moved their heads around. Thus, an image mapped on the interior of a sphere and centered on the participant was sufficient to provide the room environment used in this study. It also reduced the processor load needed to render 3D images during the experiment and reduced time lags in image generation by tracking head motions. Conversion of the 3D model to a 360-degree panoramic image was conducted with the script *Camera.RenderToCubemap* in the Unity software version 2022.3.

The test and matching stimuli comprised a tile-like object with a square shape, facing the participant (Figure 2b). The matching stimulus was presented in the room, and its luminance was adjustable via a mouse wheel in the hand of the participant. The test stimulus was placed in a viewing box. This viewing box was placed at the height of the test stimulus and presented the test illuminant environment inside. The inside of this viewing box was tiled with various lightness-level surfaces, like the room, but no furniture was placed in it. 

The Illuminance conditions were simulated by simply scaling the luminance of the background image, which was mapped on the inner wall of the spherical structure (radius = 3.0 m) that surrounded the participant, to provide an illuminant environment. The illuminant conditions are represented by the luminance ratio of the “highest” lightness, white, in each environment (Table 1). The luminance of the highest lightness, white (i.e., a 100% reflectance object under 100% illuminance conditions), was 90.4 cd/m^2^. 

The simulated illuminance of the viewing box and that of the room were controlled independently, and the illuminant conditions are represented by the luminance ratios for “white” between the room and box hereafter. The ratios between the viewing box (test environment) and the room (matching environment) were 1:4, 1:2, 1:1, 2:1, and 4:1. The 1:1(a) and 1:1(b) shown in Table 1 are the control conditions to confirm the consistency among the two room luminance levels and the precision of the participants’ settings.

The luminance of the test stimulus was 0.46, 1.62, 5.49, 10.48, or 18.22 cd/m^2^. The set of test luminance values was fixed across all illuminant conditions. These were defined by equal steps in a logarithmic scale, which roughly simulated the equal steps in the lightness scale. The perceived lightness of the test stimulus could differ, depending on the illuminance level. For example, an 18.22 cd/m^2^ surface under a 25% illuminant condition (see Table 1) corresponds to a surface with 80.6% reflectance, while the same luminance surface yields 20.2% reflectance under a 100% illuminant condition. All results were assessed on the CIE L* scale to reflect the non-linear property of human lightness perception.

The degree of lightness constancy was evaluated by the Brunswik ratio (BR) [4], defined in Equation (1). L*_match_ represents a CIE L* of matched luminance, L*_lum_ represents a CIE L* of the tested luminance under room illuminant, and L*_perfect_ represents a CIE L* when the perfect lightness constancy is obtained. For example, when 18.22 cd/m^2^ was chosen as a test luminance in the viewing box under the 4:1 condition, BR would be 100% when the matched luminance was 74.5 cd/m^2^ and would be 0% when matched only in terms of luminance (i.e., 18.22 cd/m^2^). In this example, the L*_lum_ would be 52.5, while L*_perfect_ would be 92.5:

(1) $$R=\frac{\left({L^{*}}_{match}-{L^{*}}_{lum}\right)}{\left({L^{*}}_{perfect}-{L^{*}}_{lum}\right)}$$

Since the denominator of the BR formula would be zero, it is theoretically impossible to define BRs for the control conditions. Instead, we assessed the average errors ± SDs. These were 1.68 ± 1.09 and 0.56 ± 1.72 in CIE L*, under conditions 1:1(a) and 1:1(b) in Table 1, respectively. This means that the mean of the error was smaller than the 95% confidence interval (=1.96 × SD) of matching precision under each condition. Therefore, the precision of matching by the participants was sufficiently high for the experiment.

The participants were asked to match the lightness of the test object and the matching stimuli by adjusting the luminance of the matching stimulus with a wheel on a mouse. They were allowed to move their head sideways to travel in and out of the viewing box (Figure 2b). Their head position was monitored by the HMD system, and the duration of their stay inside the viewing box was limited to 30 s; the participant’s view was forcefully switched back to the room if the time was exceeded. There was no time limitation for the total duration of each trial; they could spend as much time as they wished on adjustment until they reached a satisfactory match, but the time they could spend in the viewing box was no longer than 30 s. Each stimulus condition was presented in a pseudo-random order. Each participant had completed five repetitions of settings by the end of experiencing all conditions.

In Experiment 1, we tested lightness perception for the five test samples under six illuminant conditions (Table 1). To assess the effect of the head-tracking ability of HMDs, we conducted another experiment (Experiment 2) while disabling the head-tracking mode of the HMD. Since the apparatus was the same as that used in Experiment 1, we describe herein the parts of the methods that were different from those in Experiment 1. Participants were asked to position their heads on a chin-rest and were not allowed to move their heads. Instead, they used mouse buttons to switch their view between the two illuminant environments (the room and the viewing box) to conduct the assigned matching task, which was the same as that in Experiment 1. In this experiment, only two extreme conditions of illuminance ratios (1:4 and 4:1 in Table 1) were tested. The time that was allowed for the participants to stay in the box was limited to 30 s, as in Experiment 1. Five of the participants in Experiment 1, including the second author, participated in Experiment 2.

The third experiment tested whether spontaneous motion was the significant factor causing the high BRs in Experiment 1. In this experiment, we asked the participants to fix their heads in position during the matching, but the scene movements in the HMD were generated by the pre-recorded head trajectory of the same participant in Experiment 1. The illuminant and test conditions were the same as those in Experiment 2. Five of the participants in Experiment 1 were also involved in Experiment 2. The participants were partially different between Experiments 2 and 3, but the second author participated in all experiments.

## 3. Results

### 3.1. Experiment 1

**Figure 3:**
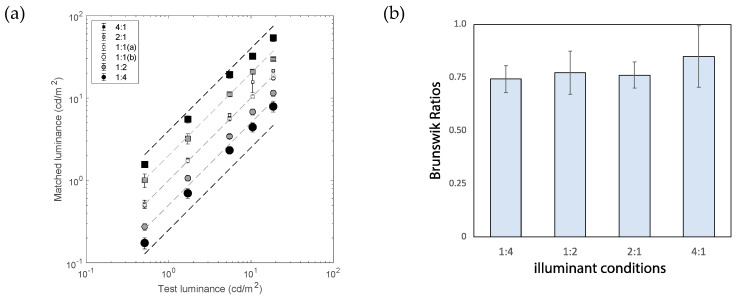
(**a**) Results of matchings between the test and matching stimuli (*N* = 11). The different symbols represent different illuminant conditions (see figure legend and Table 1). Error bars indicate the SEM. Oblique dashed lines indicate 100% constancy under 5 illuminant conditions. (**b**) Averaged constancy indices for four illuminant conditions. Error bars indicate the SD across test luminance values.

Figure 3 shows the result of Experiment 1. Figure 3a shows the results of luminance matching. The horizontal and vertical axes represent the luminance of the test and matched results, respectively. If the participants matched only in terms of luminance, the results would be aligned to the middle diagonal line, the same as in the 1:1 condition. The results for conditions 4:1 and 2:1 (square symbols) show a good alignment with the dashed diagonal lines that represent the 100% level of lightness constancy. The shading of the symbols represents the differences in the illuminant intensity conditions. The results for the 1:2 and 1:4 conditions (round symbols) fall slightly closer to the diagonal dashed lines. This finding will be assessed by using quantitative measures using the Brunswik ratios (BRs). Figure 3b shows the BRs, each of which was an average across five test luminance levels for four illuminant conditions (excluding the two 1:1 conditions). The error bars indicate a ±1 standard deviation (SD) across five test luminance levels. There was no statistically significant difference across the illuminant conditions. The lowest BR in this result was 74.2 ± 6.3% (mean ± SD) for condition 1:4, which is the result of making lightness matches of objects in the darkest room-illumination condition. The highest BR was 84.8 ± 14.5% under condition 4:1, which is the result of making lightness matches with objects in the brightest room-illumination conditions. Even the lowest BR was higher than the average BR using computer screens (approx. 65%) [4].

### 3.2. Experiment 2

**Figure 4:**
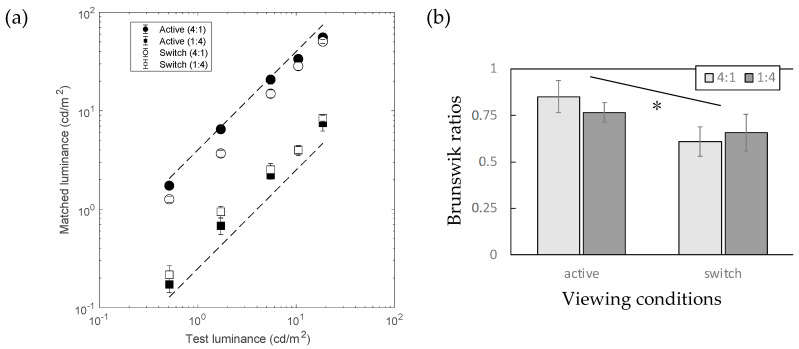
Results of Experiment 2. (**a**) Matching results under two simulated illuminant conditions (*n* = 5). (**b**) The horizontal axis represents the viewing condition and the vertical axis represents the Brunswik ratios (BRs). The different shades of bars represent the different illuminant conditions (see legends). Error bars represent SDs across the test stimulus luminance. Asterisk represents a significant difference between two conditions (*p* < 0.05; see the main text for details).

Figure 4a shows the matching results, with and without tracking the viewer’s head movements when generating images in the HMD. The scheme of Figure 4a is the same as that in Figure 3a. The open symbols represent the results under switch conditions, in which the participants used keypresses to switch their view between the room and the viewing box for matching. Two dashed diagonal lines indicate the level of 100% lightness constancy. The data for active conditions (filled symbols) are the same data as those for the illuminant condition in Experiment 1. Figure 4b shows the same results in the form of Brunswik ratios (BRs). In both the 1:4 and 4:1 illuminant conditions, the original conditions with head tracking (labeled as “active” on the horizontal axis) showed higher BRs than the conditions without head tracking (labeled as “switch” on the horizontal axis). 

A two-factor analysis of variance (ANOVA) was applied, with head tracking (active/switch) and illuminant conditions (1:4, 4:1) as factors. The results showed a significant effect of head tracking (F(1:4) = 13.1, *p* = 0.0224) and no effect of illuminant (F(1:4) = 0.252, *p* = 0.641; n.s.) conditions, but their interaction was significant (F(1,4)= 8.4689, *p* = 0.0437). A paired *t*-test within each illuminant condition also showed statistically significant differences; *t*(4) = 2.87 and *p* = 0.0455 and *t*(4) = 3.68 and *p* = 0.0211 for conditions 1:4 and 4:1, respectively. This result indicates that the effect of head motion significantly contributed to the high BRs in the first experiment.

### 3.3. Experiment 3

**Figure 5:**
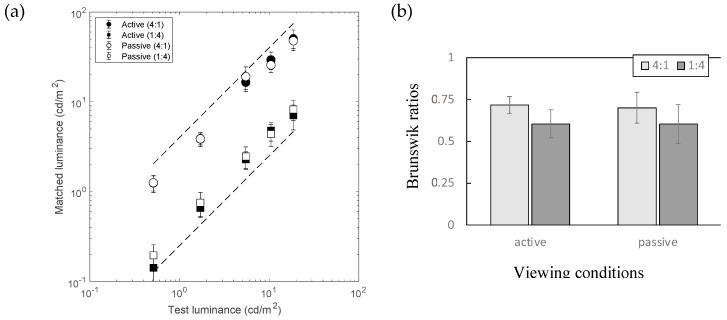
Results of Experiment 3. (**a**) Matching results under two simulated illuminant conditions (*n* = 5). (**b**) Results of Experiment 3 as BRs. The figure format is the same as that in Figure 3b.

Figure 5a shows the matching results. The scheme of Figure 5a is the same as that of Figure 3a. Open symbols represent those results under the passive condition, in which the participants conducted lightness matches while the HMD displayed a replay of the motions of the same participant in Experiment 1 in switching views between the room and viewing box for matching. Data for the active conditions (filled symbols) are the same data as those for the illuminant condition in Experiment 1. Figure 5b shows a comparison of the results between conditions with active and passive movements in BRs. The results show no significant difference in the ANOVA. This seems to suggest a consistent effect of illuminant conditions, but it was not statistically significant (F(1,4) = 2.66, *p* = 0.178) in this study.

To assess the differences between Experiments 2 and 3 directly, an additional statistical analysis was conducted by directly comparing the BRs under the switch- and passive-viewing conditions. Due to differences in the participants between Experiments 2 and 3, the overall BRs under active conditions were slightly lower in Experiment 3 than in Experiment 2 (active conditions, shown in Figure 4b and Figure 5b). To eliminate the effect of differences in this baseline performance, all the participants’ BRs in switch or passive conditions were normalized to that for active viewing conditions in each participant’s results under each illuminant condition before making any statistical comparisons. The normalized BRs for the switch and passive conditions (mean across participants ± SDs) are shown in Table 2. A two-tailed *t*-test revealed statistically significant differences between Experiments 2 and 3 under both illuminant conditions: *t*(4) = 4.05; *p* = 0.0155 and *t*(4) = 3.10; *p* = 0.0364 for 4:1 and 1:4 conditions, respectively. 

## 4. Discussion

The precision of lightness matching under control conditions was high since the average error in CIE L* was 1.68 ± 1.09 and 0.56 ± 1.72 (mean ± SD) under 1:1(a) and 1:1(b) conditions, respectively. The overall precision of the asymmetric lightness matches yielded BRs of around 75%–85%. The lowest was much higher than the average BRs for computer monitor studies (around 65% [4,6,7,8]) and the highest was at the same level as BRs for real-object studies (around 85% [4,9,10,11,12,13,14,15,16]). Although the scene articulation on the walls of the “room” was not realistic (achromatic patches), it is possible that the scene illuminant perception, which can be only indirectly measured by lightness perception, could be much higher when more realistic scene images (light fields) are used in the virtual space of HMDs.

In comparison with previous studies using computer displays, the degree of lightness constancy in our study was higher, on average, while those of color/lightness constancy when measured with real objects are similarly high [4,9,10,11,12,13,14,15,16]. Among the studies using computer screens, some studies marked a higher constancy index of over 90 percent [22]. 

The effect of head-motion tracking was evident (Experiment 2), but the motion was not necessarily made spontaneously (Experiment 3); the replay and spontaneous motion yielded no significant differences in BRs in Experiment 3, which showed significant differences from the results for the switch condition (Table 2). This implies that showing a wider field of view by presenting a moving scene gives richer clues to its observers [14,20,21]. This result is also in line with the better performance seen in a previous study on lightness/color constancy when stimuli were presented in motion [3,4,24,36,37]. A previous study has reported that the visual environments that VR goggles can present are still limited [38]; for example, the field of view is much smaller than that of natural vision at a glance when the head-tracking function is disabled. Considering the integration of lightness information over time [30], the effect of the smooth motion of the image on the HMD screen, generated by the head motions of the viewers, allows the human visual system to access much richer visual information than when using static images on flat computer displays [4,5,6,7,8,14,15,16,17,18,19,20,21,22].

It was also reported in our previous study using the HMD that the participants’ motions of head position and posture affected the performance of a visual search task, in which the participants were asked to find a target item from another viewpoint that was different from that used when the target was designated [39]. Another study suggested that the visual search performance was better (even at the back of the participant) when the 360-degree display was used, and the participant was allowed to look around by twisting their body [40]. This indicates that changes in the participants’ view, in synchrony with head motion, are important for the recognition of a three-dimensional space around the participant, and such an effect may have contributed to high performance in the lightness-matching task.

However, we did not test whether the use of motion by the same participant in passive conditions was crucial. If the result of lightness constancy is degraded by the use of the other participants’ head motion in the replay, this would suggest the effect of individual differences in the strategies used for head movement or the deployment of attention. To elucidate these factors, it is necessary to design additional experiments to address these questions, and that is beyond the scope of the present study. We would like to study this point in future experiments.

Although the effect was not statistically significant, some participants seemed to exhibit lower BRs when the room (the matching environment) illuminant was darker (25% in luminance for the whitest surface, normalized to the maximum luminance available on the HMD screen) in Experiment 3 (Figure 5b). The results of Experiment 1 initially showed a similar trend when the number of participants was small (around five), but the difference became statistically non-significant after the number of participants was increased to eleven. This implies the presence of individual differences in the asymmetry of BRs between conditions 1:4 and 4:1, probably because some participants tended to stay in darker places for longer/shorter periods than others, and that this could have caused individual differences in the state of light adaptation. Since adaptation to a darker environment takes a much longer time (in the order of several tens of minutes) to complete the adaptation than to adapt to a brighter environment (in the order of seconds), making adjustments for lightness matches in a darker room (1:4 and 1:2 conditions) could have yielded more incomplete adaptation than when the participants made adjustments in a brighter room (4:1 and 2:1 conditions). Since the recording of head-motion trajectory was preserved only for those who participated in both Experiments 1 and 3, it is impossible to make comparisons among the entire group of participants in the present study. However, it will be important to pay careful attention to and control the state of light adaptation in future studies. 

## 5. Conclusions

We have measured the degree of lightness constancy in the virtual environment presented by an HMD system. The degree of lightness constancy was as high as 75–85% in terms of the BR constancy index, which was much higher than the average constancy index of 65% reported in studies using a 2D computer screen and was as high as the average constancy index of 80% with real objects. Therefore, the quality of the virtual environment in HMDs can be as real as in real environments, even though a non-realistic wall (of tiled gray squares) was used. Therefore, the degree of lightness constancy could be much higher if a more realistic scene were used. This high degree of reality was supported by the ability of the system of HMDs to present a virtual scene in synchrony with the head motion of the viewer. However, it was not necessary that the motion, which contributed to the extension of the field of view, should be synchronous with the live motion of the viewer.

## Figures and Tables

**Table 1:** Illuminant conditions.

Illuminance Ratio	4:1	2:1	1:1(a)	1:1(b)	1:2	1:4
Room ”white” (cd/m^2^) (in percentage)	90.4 (100%)	90.4 (100%)	90.4 (100%)	22.6 (25%)	22.6 (25%)	22.6 (25%)
Box “white” (cd/m^2^) (in percentage)	22.6 (25%)	45.2 (50%)	90.4 (100%)	22.6 (25%)	45.2 (50%)	90.4 (100%)

**Table 2:** Normalized BRs (mean ± SD) in Experiments 2 and 3.

Illuminant Conditions	Switch (Experiment 2)	Passive (Experiment 3)
4:1	0.858 ± 0.112	1.03 ± 0.0918
1:4	0.72 ± 0.148	0.996 ± 0.0914

## Data Availability

The data is available at the following OSF website. https://osf.io/427bk/?view_only=77cdd0aacf90481a826f9a421e79bc1e (accessed on 10 January 2024).

## Supplementary Materials

The following supporting information can be downloaded at: https://www.mdpi.com/article/10.3390/jimaging10020036/s1, Table S1: Luminance profile of OLED screen on Oculus Rift DK2.
