# Supplementary material for: The Reality of a Head-Mounted Display (HMD) Environment Tested via Lightness Perception
Source: J Imaging. 2024 Jan 29;10(2):36. doi: 10.3390/jimaging10020036 (PMC10889787; doi:10.3390/jimaging10020036)
Supplement: Supplementary file 1 [file jimaging-10-00036-s001.zip › jimaging-2803736-supplementary.pdf]

Supplementary material 1: Luminance profile of OLED screen on Oculus Rift DK2

Top row shows RGB value ((R, G, B) = (x, x, x)) of a uniform screen and the bottom row of corresponding column shows luminance on the screen in cd/m^2, measured by a spectroradiometer SR-UL1R (Topcon, Japan). A program written in Unity was used to present an uniform rectangular tile, covering the entire OLED screen, for the luminance calibration.

**RGB val.**	**0**	**1**	**2**	**3**	**4**	**8**	**16**
Lum. (cd/m^2)	$1.46\times^{-5}$	0.0120	0.0378	0.0751	0.114	0.281	0.625
		24	32	40	48	56	64
		1.11	1.71	2.43	3.28	4.31	5.49
		72	80	88	96	104	112
		7.00	8.38	10.07	11.71	13.79	15.98
		120	128	136	144	152	160
		17.4	20.9	22.4	26.1	28.5	30.5
		168	176	184	192	200	208
		35.8	39.8	42.9	46.0	51.0	60.1
		216	224	232	240	248	255
		61.3	62.4	70.6	71.7	76.5	90.4
